# *ShF5H1* overexpression increases syringyl lignin and improves saccharification in sugarcane leaves

**DOI:** 10.1080/21645698.2024.2325181

**Published:** 2024-03-20

**Authors:** Juan Pablo Portilla Llerena, Eduardo Kiyota, Fernanda Raquel Camilo dos Santos, Julio C. Garcia, Rodrigo Faleiro de Lima, Juliana Lischka Sampaio Mayer, Michael dos Santos Brito, Paulo Mazzafera, Silvana Creste, Paula Macedo Nobile

**Affiliations:** a Department of Plant Biology, Institute of Biology, University of Campinas, Campinas, Brazil; b Academic Department of Biology, Professional and Academic School of Biology, Universidad Nacional de San Agustín de Arequipa, Arequipa, Perú; c Centro de Cana, Instituto Agronômico (IAC), Ribeirão Preto, Brazil; d Institute of Science and Technology, Federal University of São Paulo, São José dos Campos, Brazil; e Departamento de Genética, Faculdade de Medicina de Ribeirão Preto, Universidade de São Paulo, Ribeirão Preto, Brazil

**Keywords:** Bioenergy, ferulate 5-hydroxylase, lignocellulose, monolignol, saccharum spp

## Abstract

The agricultural sugarcane residues, bagasse and straws, can be used for second-generation ethanol (2GE) production by the cellulose conversion into glucose (saccharification). However, the lignin content negatively impacts the saccharification process. This polymer is mainly composed of guaiacyl (G), hydroxyphenyl (H), and syringyl (S) units, the latter formed in the ferulate 5-hydroxylase (F5H) branch of the lignin biosynthesis pathway. We have generated transgenic lines overexpressing *ShF5H1* under the control of the *C4H* (cinnamate 4-hydroxylase) rice promoter, which led to a significant increase of up to 160% in the S/G ratio and 63% in the saccharification efficiency in leaves. Nevertheless, the content of lignin was unchanged in this organ. In culms, neither the S/G ratio nor sucrose accumulation was altered, suggesting that *ShF5H1* overexpression would not affect first-generation ethanol production. Interestingly, the bagasse showed a significantly higher fiber content. Our results indicate that the tissue-specific manipulation of the biosynthetic branch leading to S unit formation is industrially advantageous and has established a foundation for further studies aiming at refining lignin modifications. Thus, the *ShF5H1* overexpression in sugarcane emerges as an efficient strategy to improve 2GE production from straw.

## 1. Introduction

Sugarcane (*Saccharum spp*.) is one of the most efficient crops converting solar energy into photoassimilates, accumulating up to 18% sucrose in its culms^1^ and used as an excellent feedstock for sugar and bioethanol production.^2,3^ The yield of sugarcane dry biomass expressed as unit per area (39 Mg/ha, culm, leaves, and top) is significantly higher than maize (17,6 Mg/ha, grain and straw), *Panicum* (10,4 Mg/ha; biomass), and *Miscanthus* (29,6 Mg/ha; biomass).^4,5^ During the mechanical sugarcane harvest, the straw, consisting mainly of dry and green leaves and the tip of the culm, is usually left in the field, improving soil properties and crop productivity.^6^ However, recent research has shown that it can be partially removed and used for other purposes without significant productivity losses.^7^ The sugarcane straw represents a potential feedstock for cellulosic ethanol, also named second-generation ethanol (2GE), and has an energy potential of about 30% of the total biomass conversion into ethanol.^8^

Lignocellulosic biomass has a complex architecture and composition that prevents its prompt conversion into 2GE. The cellulose microfibril grid is embedded in a matrix of hemicellulose covalently attached to lignin.^9^ Lignin is an aromatic polymer that plays a pivotal role in plant growth, particularly in xylem water transport.^10^ Additionally, cell wall robustness confers recalcitrance to the cellulose conversion into glucose (saccharification).^11,12^ The highly laborious and time-consuming genetic breeding program in sugarcane and the little associations between agronomic traits and cell-wall components^13^ make genetic engineering of the lignin metabolic pathway an attractive alternative. Biotechnological manipulation aims to change the content and composition of lignin in plant tissues by functional validation and modulation of the genes involved in the biosynthesis, regulation, and polymerization of this molecule in different plant genera.^14^ The degree of reduction and alteration of lignin composition depends on the enzyme position in the biosynthetic pathway, the target gene, and the genetic manipulation strategy, i.e., overexpression, silencing, and mutation.^14,15^

Lignin is formed by the polymerization of three canonical monomers, syringyl (S), guaiacyl (G), and *p*-hydroxyphenyl (H), with possible incorporation of non-canonical and unusual phenylpropanoid intermediates.^16–18^ For instance, in grasses, the γ-*p*-coumaroylated monolignols, namely coniferyl and sinapyl *p*-coumarates, give rise to the γ-*p*-coumaroylated G and S lignin units, which incorporate into the lignin together with the S and G units common to eudicots.^19–21^ The proportions of the monomers affect the physical structure of lignin and, therefore, the digestibility of the cell wall.^22,23^ The difference in the methoxylation degree of coniferyl alcohol (monomethoxylated) and sinapyl alcohol (dimethoxylated) causes G-lignin to be more branched than the relatively linear S-lignin.^24^ Knowledge about the S/G ratio and the intermonomer linkage in the lignin polymer are relevant to predicting the degree and nature of polymer reticulation.^25^ The S/G ratio impact on biomass digestibility has been investigated using different approaches: *i*. natural genetic variation,^25–28^ *ii*. artificial formation of the secondary wall using cells with primary walls as a model,^29^ and *iii*. genetically modified plants.^29–31^ Such genetic manipulations resulted primarily in the ratio change of the canonical monomers [p-hydroxyphenyl (H), guaiacyl (G), and syringyl (S)].^31^ Altogether, most reports showed that the S/G ratio and digestibility are correlated.

Two enzymes are closely related to the S units biosynthesis branch, ferulate-5-hydroxylase/coniferaldehyde 5-hydroxylase (F5H/Cald5H) and the caffeic acid 3-*O*-methyltransferase (COMT). F5H catalyzes the conversion of coniferyl aldehyde to 5-hydroxyconiferyl aldehyde^32^ and COMT preferably catalyzes the methylation of 5-hidroxyconiferaldehyde and/or alcohol 5-hydroxyconiferil to form synapaldehyde and sinapyl alcohol, respectively.^33^ Conifers (softwoods) naturally lack syringyl units in their lignins and the heterologous expression of *F5H* and *COMT* in *Pinus radiata* led to the incorporation of the S unit in the tracheary element system.^34^ Both enzymes have been targeted for genetic engineering via interference RNA (RNAi) and/or mutant genotypes to increase G-units and decrease the S/G ratio, enhancing biomass digestibility.^30,34–39^

Improved biomass digestibility by genetic manipulation often results in plant yield penalties.^40^ Although biomass rich in S-lignin is associated with higher biomass digestibility, *F5H* overexpression has been little explored. Poplar^30^ and Arabidopsis^40,41^ showed enhanced digestibility when the S/G ratio was increased by *F5H* overexpression. In monocots, F5H was identified and characterized in rice,^42^ switchgrass,^31^ sorghum^43^ and barley,^44^ and its counterpart function on the S branch was confirmed but still lacked a demonstration of digestibility improvement. It has been reported that CAld5H (coniferaldehyde 5 hydroxylase) has a different activity compared to F5H as the former is more specific to the downstream pathway.^45,46^

Although most investigated plants share similarities in the lignin biosynthesis pathway,^47^ information on sugarcane is limited due to its high polyploidy and complex genome, not yet fully sequenced because difficulty in assembling a reference genome.^47,48^ Modern cultivars are highly polyploids, having 2n = 100–120 chromosomes.^49^ They are hybrids from the crossing between *Saccharum officinarum* (2n = 80) and *Saccharum spontaneum* (2n = 40–128). Only one consensus sequence of *F5H* transcript was identified in commercial cultivars differing in lignin content^50^ and in the species *S. officinarum*, *Saccharum barberi*, *S. spontaneum*, and *Saccharum robustum*,^51^ showing a conserved sequence in the genus.

Despite its relevance as a bioenergy crop, only some reports on the manipulation of lignin metabolism in sugarcane are available in the literature (see Table S1). These reports evaluated the impact of suppressing different genes (*ShCOMT*, *ShCCoAOMT*, *ShF5H1*, and *4CL*) on lignin and saccharification. The downregulation (up to 84%) of *F5H* reduced the S/G ratio from 1.56 in the wild type to 0.92 in the transgenic lines, did not change the lignin content, and improved up to 31% the bagasse saccharification efficiency^35^ (Table S1). To our knowledge, neither the manipulation of lignin metabolism in sugarcane leaves nor the strategy of overexpressing *F5H* to increase the S/G ratio were investigated for this species. Sugarcane leaves have a 2–3 fold lower S unit in lignin composition than culms,^25,51^ which allows investigating whether the increase in S-lignin influences saccharification efficiency.

In this study, the *ShF5H1* gene from the *Saccharum* hybrid IACSP04–065 was overexpressed under the control of the C4H (cinnamate 4-hydroxylase) rice promoter in the commercial sugarcane cultivar SP80–3280. Biochemical analyses (cell wall composition, saccharification efficiency, and total soluble phenols) revealed a significant increase in S/G ratio and saccharification efficiency in the transgenic lines without alternations in the lignin content.

## 2. Material and Methods

### 2.1. Sequence Analysis and Construction of the Expression Cassette

*ShF5H1* full-length coding DNA sequence (CDS) (GenBank access number KT777465.1) was isolated from the fifth and sixth internodes of the *Saccharum* hybrid IACSP04–065 genotype via reverse-transcriptase polymerase chain reaction (RT-PCR). The pair primers (Table S2) were designed from partial sequences retrieved from a sugarcane RNA-seq database produced in our lab^52^ and from the SUCEST database (http://sucest-fun.org) (SCJLRT1022E04 g).

*ShF5H1* was first translated into the amino acid sequence and homolog sequences were searched using Basic Local Alignment Search Tool (BLASTp) 2.2.8 in the Phytozome v12.1 and v.13 (*Saccharum officinarum x spontaneum* R570 v2.1), and the *Arabidopsis* Information Resource (TAIR BLAST 2.9.0+). The protein sequences were retrieved from *Sorghum bicolor* (Sb), *Zea mays* (Zm), *Oryza sativa* (Os), *Panicum virgatum* (Pv), and *Arabidopsis thaliana* (At). Additionally, previously reported sequences were included in the phylogenetic analysis: PvF5H1a (GenBank accession number BAO37287.1)^31^ and barley (Hv), wheat (Ta), *Brachypodium* (Bd), *Miscanthus* (Ms), green foxtail (Sv), *Selaginella moellenodorffii* (Sm).^44^ The amino acid sequences (aa) were aligned in the ClustalX v2 program.^53^ The evolutionary history was inferred using the Maximum Likelihood method and Whelan and Goldman model.^54^ Initial tree(s) for the heuristic search were automatically obtained by applying Neighbor-Join and BioNJ algorithms to a matrix of pairwise distances estimated using the JTT model and then selecting the topology with a superior log-likelihood value. A discrete Gamma distribution was used to model evolutionary rate differences among sites [5 categories (+G, parameter = 1.3116)]. The tree was drawn to scale, with branch lengths measured in the number of substitutions per site. Evolutionary analyses were conducted in MEGA X.^55^

The promoter region of the rice cinnamate 4-hidroxylase gene - *C4H*^56^ was selected to control the expression of *ShF5H1* in the plant cassette. The fragment with 2068 pb from genomic DNA of rice (*O. sativa* L. var. Nipponbare) was isolated using Os05g25640 sequence available in the Phytozome database (http://phytozome.jgi.doe.gov/pz) as reference. The forward primer was designed harboring upstream the start codon (ATG), and the reverse primer was designed 2070 pb downstream of the ATG start codon, comprising TATABOX consensus and UTR 5.’ The PCR product was cloned and sequenced. The alignment among the cloned sequence and reference sequences from GenBank and Phytozome had 100% identities, showing a gap with two nucleotide cytosine deleted in the 1846 and 1847 pb positions (Figure S1).

The *ShF5H1* fragment was cloned into pDONR221 vector using the Gateway Recombination System™ (Invitrogen Life Technologies, US) following the manufacturer’s instructions. Through multiple recombination reactions using pK7m24GW.3 as backbone vector (Flanders Interuniversity Institute for Biotechnology, Universidad de Ghent, Belgium - https://gateway.psb.ugent.be/), a cassette containing *ShF5H1* coding DNA sequence (CDS) fused to rice promoter C4H and the Cauliflower Mosaic Virus (CaMV) 35S terminator (*T*-35S) was generated (Figure S2). Next, pK7m24GW.3/pOsC4H:*ShF5H1* vector was subject to digestion with the restriction enzymes BamHI and HindIII, releasing the pOsC4H:*ShF5H1* cassette. Then, the cassette was ligated to the final binary vector pJFnptII,^57^ kindly provided by Prof. Fredy Altpeter (Agronomy Department, Genetics Institute, University of Florida, IFAS, Gainesville, FL, USA), containing the nptII selection cassette (pZmUbi-nptII-T-35S). The pJFnptII-pOsC4H:*ShF5H1* vector was cloned into *Agrobacterium tumefaciens* AGL1 cells for sugarcane genetic transformation.

### 2.2. Plant Material and Genetic Transformation

Plants of the commercial sugarcane hybrid SP80–3280 were grown for 6 to 9 months in 50 L pots in a greenhouse (Sugarcane Center, Agronomic Institute of Campinas, Ribeirão Preto-SP, Brazil). Immature leaf roll explants were processed according to^58^ and inoculated into a modified sugarcane callus induction medium (SCIM_3_) (Table S3). Thirty grams of *calli* were used for genetic transformation via *Agrobacterium*, according to Dong et al.^59^ and Basso et al.^58^ In parallel, we also separated three grams of *calli* to carry out all the steps of the transformation protocol and suppress *A. tumefaciens* to generate non-transformed *calli*, which we used as control. Three main stages of *in vitro* tissue culture were carried out in Murashige and Skoog (MS) Basal Medium (Table S3): *1*. selection and multiplication of cells in the dark; *2*. shoot regeneration; *3*: elongation and rooting. Rooted plantlets were acclimatized in the greenhouse for two weeks at 80% relative humidity and 80% shadow. Non-transformed plantlets (“control”) were subjected to the same tissue culture stages of transformed plantlets, except for the contact with the selective agent (G418).

Control and G418 selected plantlets were grown under greenhouse conditions in 750 ml pots containing a mixture (1:1, v/v) of soil and substrate (Carolina Soil, Santa Cruz do Sul, Brazil) for three months. From an initial *ShF5H1* transcriptional analysis, we selected nine independent events for further study. These T0 plants were transferred to 18 L pots using the same substrate mixture and remained in greenhouse conditions for nine months, each pot containing three tillers per clump. First-generation (T1) *ShF5H1* transgenic lines were vegetatively propagated by pre-sprouted sets, which were small sections obtained from one-bud sets. Three propagules from each transgenic line (L4, L6, L49, and L58) and four from control lines were planted in 50 L pots and kept in the greenhouse for nine months. Maturity (sucrose accumulation) was induced by irrigation suspension for five days.

For molecular and biochemical analyses, + 1 leaves were harvested from T0 plants grown in the greenhouse for 45 days, ~4 months, and ~7 months, and from T1 plants grown in the greenhouse for nine months, with 10 cm (45-day-old plants) and 30 cm (adults) length of leaf blade from the base to apex. Mature internodes (8^th^) of T1 plants, counting from the apex of the culm, were harvested for gene expression and S/G ratio analysis.

### 2.3. Molecular Analysis

The quantitative PCR (qPCR) reactions were performed in the Applied Biosystems StepOnePlus System (Foster City CA, USA) in a final volume of 10 μL using GoTaq qPCR Master Mix (Promega, Fitchburg, WI, USA). PCR efficiency of all primers used in this study (>90% < 110%) was estimated by LinReg Software.^60^ Specific pairs of primers are listed in Table S2. Three technical replicates were used for each sample and negative controls (reactions without DNA or cDNA templates).

The presence of T-DNA in 45-day-old plantlets (T0) growing in the greenhouse was confirmed by qPCR. Genomic DNA from leaves of 60 plantlets and four controls was isolated according to.^61^ The forward primer was designed to be anchored in CaMV 35S terminator and the reverse primer was anchored in the *ShF5H1* CDS (Table S2). The positive transgene detection was evaluated by the melting curve yield in transgenic events compared to the melting curve of positive (plasmid DNA pJFNPII-pOsC4H:F5H) and non-transgenic control plants. The non-transgenic control plants showed only a background of fluorescence with a residual melting curve from unspecified amplification of primers dimers like negative control reactions. In addition, the cycle threshold (Ct) values were considered to confirm a cutoff for positive samples.

Total RNA from leaves of 45-day-old (T0) and 9-month-old (T1) plants was isolated using TRIZOL reagent (Invitrogen, Carlsbad, CA, USA). Total RNA from the 8^th^ internode of the 9-month-old primary culms was isolated according to.^62^ The RNA was treated with DNase (Promega, Fitchburg, WI, USA) to eliminate contamination with genomic DNA, and its concentration was determined with NanoDrop 2000 (Thermo Fisher Scientific, Wilmington, DE, USA).

The relative expression (RE) was evaluated in leaves and calculated using REST © software, by performing the statistical model Pair-wise Fixed Reallocation Randomization Test © with 2000 interactions.^63^ The 8^th^ internode RE analysis was calculated via the 2^−ΔΔCT^ method.^64^ In both analyses, the non-transformed plants from tissue culture (control) were used as a calibrator, and glyceraldehyde 3-phosphate dehydrogenase (GAPDH) was used as a reference gene^50^ (Table S2).

### 2.4. Growth Measurements, Juice Sugar Content, and Fiber Content of the Culm

The clumps of the 9-month-old transgenic lines and control plants were cut off from the pots. Growth measurements were evaluated using a caliper, ruler, measuring tape, and hook scale to assess: *i*. length (m), tiller diameter (cm), and internode number; *ii*. length (m), width (cm), and leaf number; *iii*. weight (kg) of the aerial part (the clumps). The leaves and the tips were removed from the culms. Then, the culms were milled, generating the moist cake. The weight of the moist cake (WMC) was recorded and then subjected to compression to extract the juice. The total soluble solids (Brix), apparent sucrose (POL), sucrose, purity, POL broth, and reducing sugars of juice, as well as the moisture and the total fiber of the dry cake (bagasse) were determined according to the methods recommended by.^65^

### 2.5. Biochemical Characterization of T1 Transgenic Plants

Leaves and 8^th^ internodes were powdered in liquid nitrogen and freeze-dried. The lignin content (% cell wall) was determined by the acetyl bromide method^66^ using 20 mg of leaves from 9-month-old plants.

Lignin monomers were determined in the leaves of T0 (4-month-old plants) and T1 transgenic lines (9-month-old plants), and in the 8^th^ internodes (T1). The samples were digested with NaOH at 95°C/24 h, neutralized with HCl, and extracted with ethyl acetate. The residue was dried and then solubilized in H_2_O MilliQ, and the hydrolysis products were analyzed by LC-MS using a UHPLC coupled to a triple quadrupole mass spectrometer with ESI ionization source (model ACQUITY, Waters Corp., Manchester, UK), as described by.^67^ Analytical external calibration curves were built by injecting each standard solution in triplicate and used to calculate each monomer. Cellulose was extracted from the leaves and internodes according to,^68^ with modifications described by.^51^

Saccharification was determined according to^69^ with minor modifications, using lyophilized biomass equivalent to 10 mg of cellulose.^51^ In microtubes, an amount of lyophilized biomass equivalent to 10 mg of cellulose (previously determined, according to the protocol above and free of soluble sugars and starch) was weighed, and added 500 µl of sodium citrate buffer (0.1 M, pH 4.8), 10 µl of sodium azide and water to 1 ml. Subsequently, the mixture was heated to 50°C and added 6.08 µl of a mixture (1:4, v/v) of cellulase enzymes (1.2 FPU/10 mg of cellulose) and cellobiose (1 .26 U pNPGU/10 mg cellulose). The cocktail was prepared from *Aspergillus niger* cellobiose and *Trichoderma reesei* cellulase (Sigma-Aldrich). The microtubes containing the samples and the reaction solution were hermetically closed and placed at an angle (45°) on an orbital shaker at 50°C, maintained at 160 rpm for five days. After, the samples were centrifuged at 12,000 rpm for 15 min. Glucose determination was carried out in the supernatant by the sulfuric phenol method.^70^

Phenolic profiling was carried out by ultra-performance liquid chromatography coupled to a mass spectrometer, according to,^71^ with modifications. Lyophilized (50 mg) leaves from 9-month-old T1 plants were extracted with 500 µL ethanol:H_2_O (4:1, v/v). The mixture was vortexed for 30 s, sonicated for 10 min, and shaken for 2 h at room temperature. After centrifugation (10,000 rpm, 15 min), the supernatant was collected and dried using a centrifugal vacuum evaporator. The same volume of ethanol:H_2_O (4:1, v/v) was added to the pellet and the procedure was repeated. Dried sugarcane extracts were solubilized in 600 µL ethanol:H_2_O (1:1, v/v) and filtered through a 0.2 µm syringe filter (PTFE Millex-LG, Merck). For total soluble phenols, samples were extracted twice with 80% ethanol and the phenol content was determined with the Folin-Ciocalteu reagent,^72^ using chlorogenic acid as standard.

### 2.6. Anatomical Analysis

The plant material, SP80–3280 (control) and the L6 *ShF5H1* transgenic line (13 months old), was cultivated by Nuseed Brasil company in field trials located at Barra de São Miguel, Alagoas, with coordinates 9.817666°S (latitude) and 35.956677°W (longitude). This area was designated for Planned Release of Material into the Environment. The 8^th^ internodes of the culms were separated and sent to the Plant Anatomy Laboratory of the Department of Plant Biology at the Institute of Biology of the State University of Campinas, Campinas-SP. Samples from the mid-region of the 8^th^ internode of the culm were fixed in FAA 50 for 48 hours^73^ and subsequently stored in 70% ethanol solution. For light microscopy analyses, each sample was sectioned using a Leica SM 2010 R sliding microtome with a thickness of 18–20 µm. Subsequently, the obtained sections were clarified with sodium hypochlorite for 5 minutes, underwent distilled water baths, and were stained with Alcian Blue for 30 seconds.^73^ Portions of the sections were subjected to the following histochemical tests for lignin detection: Mäule reagent and Phloroglucinol-HLC. All sections were mounted between a slide and coverslip, with result documentation carried out by capturing images using an Olympus DP71 video camera attached to an Olympus B× 51microscope.

### 2.7. Statistical Analysis

Growth measurements, juice sugar content, fiber content of the culm, and biochemical data of leaves from T1 transgenic lines and non-transformed plants (control) were evaluated using variance analysis (ANOVA). When a significant variation was found, the comparison of means was evaluated by Tuckey posthoc test (*p* ≤ .05), using Statistics 10.0.^74^ We used three and four biological replicates for transgenic lines and control, respectively. Data were expressed by the mean ± standard error (*n* = 4 or 3).

## 3. Results and Discussion

### 3.1. *Phylogenetic Analysis of* ShF5h1

The blastp search in the sugarcane genome database *S. officinarum* x *spontaneum* R570 v2.1 (Phytozome 13) allowed us to retrieve the top sixteen protein sequences (e-values: 0 - 18e-180), compared to the previously identified^50,52^ and cloned (KT777465.1) ShF5H. The amino acid (aa) sequences of sugarcane, monocot species, and Arabidopsis, retrieved from the Phytozome and TAIR databases using *ShF5H1* as a query (BLASTp), along with the sequences from Shafiei et al.,^44^ were aligned and used to construct the phylogenetic tree (Figure S3). The CYP84 family comprises cytochrome P450 (CYP) monooxygenase enzymes, as F5H from the lignin pathway.^75^ The phylogenetic tree includes the outgroup of flavonoid 3’ monooxygenase, the outlier SmF5H, and the CYP84 family clade. The CYP84 clade forms four main groups. The most distant group consists of sorghum Sobic.002g029500, MsF5H4, OsF5HL, HvF5H5, and the TaF5H5 genome variant proteins. The second group includes the Arabidopsis protein, AtF5H1, which is functionally characterized and involved in S-lignin biosynthesis,^76^ and AtF5H2. The third group consists of OsF5H2 and BdF5H2–3. The major group is divided into two subgroups: one containing non-characterized proteins, which includes TaF5H2–3, HvF5H4, MsF5H3, SbF5H2, and ShF5H2–4. In contrast, the red-colored subgroup comprises grass proteins that have been functionally characterized (indicated by single asterisks), such as SbF5H1,^43^ OsF5H1,^42^ HvF5H1,^44^ and PvF5H1a,^31^ suggesting that the members of this subgroup play a similar role in S-lignin synthesis. Among the sugarcane CYP84A proteins, only one member belongs to the mentioned subgroup, *ShF5H1* (indicated by double asterisks), positioned closest to SbF5H, whose overexpression led to an increase in syringyl acid and S units in sorghum.^43^ *ShF5H1* exhibited six variants with identities ranging from 98.3% to 99.6%, of which one sequence was cloned (double asterisks), and five sequences were retrieved from Phytozome 13 (SoffiXsponR570-05Ag184700, 01Fg002800, 1Z014100, 01Ag202200, 01Bg183300S, Eg197600). ShF5H2 showed one variant (SoffiXsponR570.10Bg128600), ShF5H3 exhibited three variants with identities ranging from 97.6% to 99.7% (SoffiXsponR570- 10US89G086400S, 10Ag093800, 10Bg12870), and ShF5H5 had five variants with identities ranging from 96.4% to 99.6% (SoffiXsponR570 – 10Dg067400, 10Ag092100, 10Cg072900, 10os1g031500, 10Eg013700).

### 3.2. *Screening of T0 Events to Obtain* ShF5h1 *Transgenic Lines with Modified Lignin Composition in the Leaves.*

**Figure 1.**
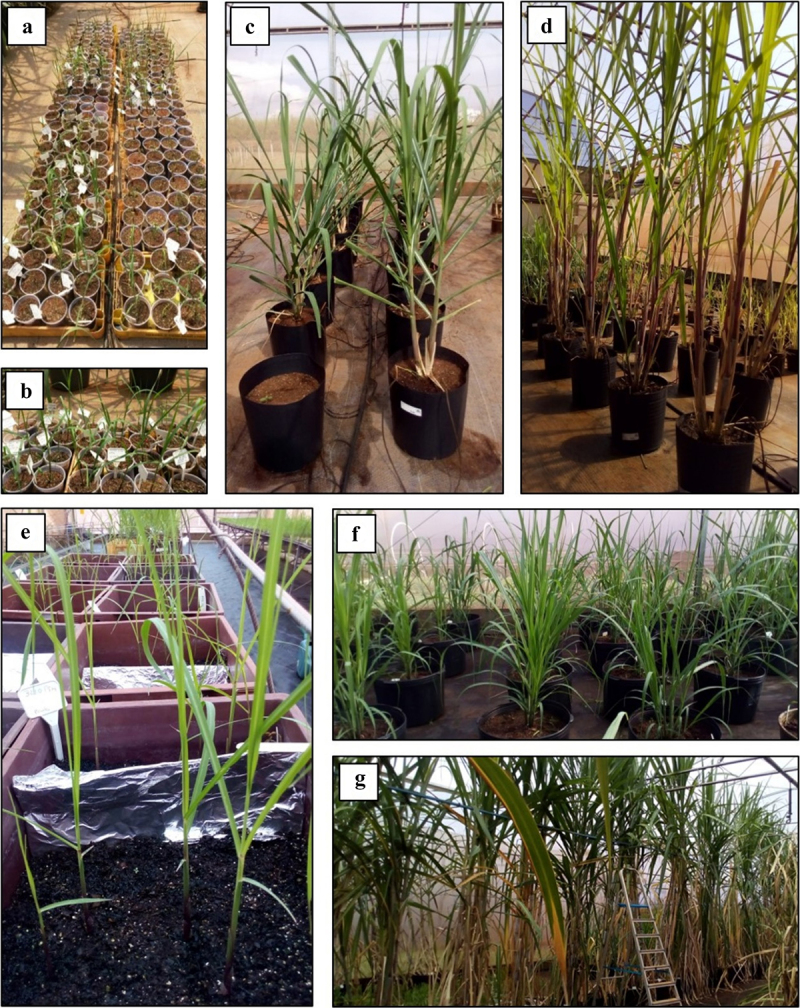
Growth of *saccharum ssp* hybrid cv. SP80–3280 transgenic lines and control in the greenhouse. (A-D) primary transformants (T0) of independent events and control. A-B. 45-day-old. C. ~4-month-old. D. ~7-month-old. T1 transgenic lines (L4, L6, L49 and L58) and control (E-G). E. pre-sprouted sets. F. ~4-month-old. G. ~9-month-old.

A total of sixty shoots were regenerated from transformed *calli* of sugarcane hybrid SP80–3280 in a selective medium containing the antibiotic G418. Each regenerated shoot was considered an independent event (Figures S4A–C). The promoter of *O. sativa* C4H was cloned into pOsC4H:*ShF5H1* expression cassette to control the specific F5H expression in lignifying tissues.^56^ The pOsC4H:*ShF5H1* was detected in 59 primary transformants (T0) from independent events (transgenic lines). The fluorescence detection obtained by the Ct values in qPCR reactions suggested that our transgenic lines have different transgene copy numbers (Table S4). Thirty-one leaves from 45-day-old T0 transgenic lines (Figure 1(a,b)) were evaluated for *ShF5H1* expression. We found a large spectrum of transcript abundance, from overexpression to downregulation, when compared to control leaves (Figure S5A). Nine of these transgenic lines (L4, L5, L6, L28, L30, L33, L49, and L58) were selected, in which the *ShF5H1* transcript levels were up-regulated (15- to 160-fold compared to the control) (Table 1). Next, these lines were planted in 18 L pots maintained at the greenhouse (Figures 1(c)). The leaves of ~ 4-month-old plants (Figure 1(d)) were evaluated to address the impact of the *ShF5H1* overexpression on the lignin S/G ratio. The S/G ratio was higher in T0 transgenic lines (from 0.32 to 0.58, Figure S5B) than control plants (0.27 on average, Figure S5B, see also Table 1). These results provide a foundation to functionally confirm that *ShF5H1* acts as an enzyme responsible for increasing the S unit levels, suggesting its role in the conversion of coniferyl aldehyde to 5-hydroxy-coniferyl aldehyde through hydroxylation of the phenyl ring.^32^

**Table 1.** *ShF5H1* transcripts analysis, guaiacyl (G) and syringyl (S) ratio (S/G), and saccharification efficiency for nine *ShF5H1* transgenic lines (T0). The columns show S/G ratio and the percentage compared to control (%/ctr); relative expression (RE) of *ShF5H1* transcripts, in which control plants were used as a calibrator; saccharification efficiency (SAC, %), and the SAC % difference compared to control (%/ctr).The S/G **(%**_**/ctr**_) and SAC **(%**_**/ctr**_) data were calculated according to the formula: (N_TL_- N_C_)*100/N_TL_+N_CTR_, where N_TL_ = S/G or SAC % of transgenic line and N_CTR_ = S/G ratio or SAC % of control plants. Lines highlighted in gray show the transgenic lines selected for cloning propagation (T1).

Lines	RE^1^	S/G^2^	S/G (%_/ctr_)^2^	SAC (%)^3^	SAC (%_/ctr_)^3^
L1	20.1	0.35	31	NA	NA
L4	35.0	0.54	103	48.3	53
L5	61.0	0.39	46	NA	NA
L6	160.6	0.58	118	52.6	66
L28	27.5	0.36	35	NA	NA
L30	146.9	0.55	106	NA	NA
L33	82.7	0.32	20	NA	NA
L49	120.6	0.45	69	51	61
L58	15.1	0.36	35	NA	NA
CTR	1	0.27	….	31.6	….

NA- not analyzed; ^1^45 day-old seedlings in the greenhouse; ^2^112 day-old young plants in the greenhouse; ^3^224 day-old adults plants in the greenhouse.

Preliminary but promising data showed that leaves of 7-month-old T0 transgenic lines (L4, L6, and L49) displayed higher saccharification efficiency compared with the control (Table 1). L4, L6, L49, and L58 transgenic lines were selected to be vegetatively propagated and for further evaluation of T1 plants.

### 3.3. Transgenic T1 Lines Showed Alterations in Growth and Culm Fiber Content.

Four transgenic lines were vegetatively propagated from bud sets of T0 plants (Fig. 1e). T1 lines L4, L6, L49, and L58 and four control plants were cultivated in 50 L pots in the greenhouse for evaluating growth, juice sugar content, and culm fiber content at nine months (Fig. 1f,g). We could not statistically differentiate T1 lines and control plants regarding leaf growth parameters (Table 2). Furthermore, no significant differences were observed in the culm diameter, number of internodes, and culm length (except for L49). L49 had a significantly smaller number of tillers than the control (*p* = .031). The weight of the aerial parts was used to infer their biomass yield, revealing that L49 showed a weight reduction when compared to the control (*p* = .0089).

**Table 2.** Growth parameters of 9-month-old T1 transgenic lines (L4, L6, L49, and L58) and control plants. Significant differences (ANOVA followed by Tukey´s test, *p* < .05) among treatments are indicated by letters. Data are means ± standard error (*n* = 3). n. = numbers.

	Culm				Leaves			Shoot
	Length (m) *p* = .037	Diameter (cm) *p* = .082	Tiller number *p* = .031	Internode number *p* = .97	Length (m) *p* = .07	Width (cm) *p* = .19	Leaf number *p* = .75	Weight (kg) *p* = .0089
L4	3.0 ± 0.21^ab^	2.4 ± 0.21	6.3 ± 0.58^a^	16.3 ± 0.58	1.3 ± 0.19	5.6 ± 0.72	7.7 ± 1.16	6.2 ± 3.33^ab^
L6	3.4 ± 0.11^a^	2.6 ± 0.06	7.0 ± 1.00^a^	16.0 ± 1.00	1.5 ± 0.10	4.9 ± 0.36	10.3 ± 1.16	9.4 ± 1.89^a^
L49	3.0 ± 0.17^b^	2.2 ± 0.23	4.0 ± 1.00^b^	14.7 ± 2.08	1.6 ± 0.16	5.1 ± 0.10	10.3 ± 2.89	4.3 ± 0.94^b^
L58	3.2 ± 0.22^ab^	2.4 ± 0.27	8.0 ± 1.00^a^	15.0 ± 1.00	1.5 ± 0.12	4.9 ± 0.12	8.7 ± 1.16	9.3 ± 1.33^ab^
Control	3.5 ± 0.17^a^	2.7 ± 0.15	6.5 ± 0.58^a^	16.3 ± 2.00	1.5 ± 0.03	5.4 ± 0.29	8.5 ± 1.15	10.5 ± 1.20^a^

Interestingly, we did not find any significant difference regarding the total soluble solids (Brix), apparent sucrose (POL), sucrose %, reducing sugars, and purity of the juice (Table 3). In contrast, the weight of the moist cake (*p* = .02) and the culm fiber content (*p* = .02) were significantly higher in the transgenic lines than in the control, especially for L6 (Table 3). This may be advantageous to produce 2GE from bagasse and its use for energy co-generation in sugarcane mills.

**Table 3.** Sugar and fiber content analyses of 9-month-old culms from T1 transgenic lines (L4, L6, L49, and L58) and control plants. Significant differences (ANOVA followed by Tukey´s test, *p* < .05) among treatments are indicated by letters. The evaluated parameters were total soluble solids (brix), apparent sucrose (POL), purity of the juice, and fiber and weight of moist cake (WMC) in the culm. Data are means ± standard error (*n* = 3).

	BRIX (% juice)	POL (%)	Sucrose (%)	WMC	POL broth (%)	Fiber	Purity	POL sugarcane	Reducing sugars	Moisture
	p = .35	p = .19	p = .19	p = .02	p = .18	p = .02	p = .069	p = .2	p = .062	p = .18
L4	15.0 ± 1.5	42.6 ± 6.9	43.0 ± 6.9	120.2 ± 3.2^ab^	10.6 ± 1.6	10.5 ± 0.3^ab^	70.3 ± 3.9	9.2 ± 1.5	1.07 ± 0.1	76.5 ± 1.2
L6	16.1 ± 1.5	50.3 ± 7.6	50.7 ± 7.6	122.8 ± 1.4^a^	12.4 ± 1.8	10.7 ± 0.1^a^	76.7 ± 4.5	10.7 ± 1.5	0.87 ± 0.1	75.4 ± 1.4
L49	15.4 ± 0.3	46.3 ± 1.1	46.7 ± 1.1	132.2 ± 9.4^ab^	11.5 ± 0.3	11.5 ± 0.8^ab^	74.4 ± 0.7	9.8 ± 0.3	0.93 ± 0.1	75.4 ± 0.6
L58	16.5 ± 0.2	52.6 ± 1.9	53.0 ± 1.9	124.1 ± 5.0^ab^	12.9 ± 0.5	10.8 ± 0.4^ab^	78.1 ± 1.8	11.2 ± 0.4	0.83 ± 0.1	74.9 ± 0.4
Control	15.4 ± 0.8	45.8 ± 4.3	46.2 ± 4.3	116.4 ± 2.9^b^	11.3 ± 1.0	10.2 ± 0.2^b^	73.6 ± 3.2	9.9 ± 0.9	0.97 ± 0.1	76.4 ± 0.8

### 3.4. Increased S/G Ratio in Leaves of T1 Lines Resulted in Higher Saccharification

#### 3.4.1. Cell Wall Composition

**Figure 2.**
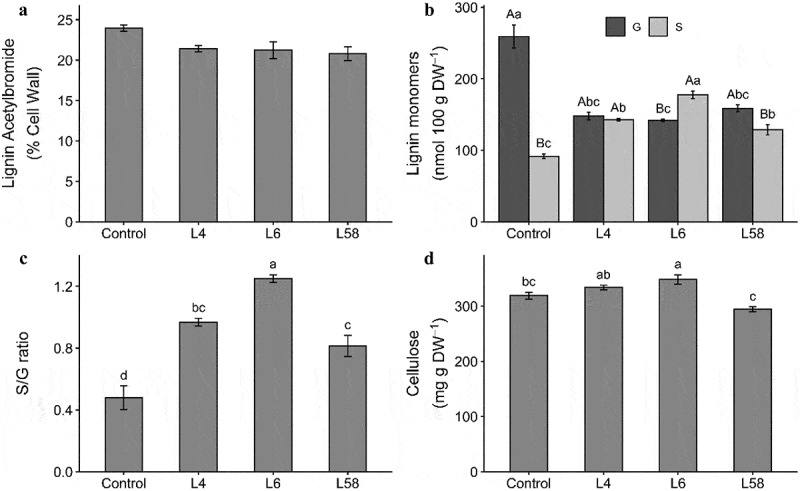
Cell wall characterization of leaves from T1 transgenic lines (L4, L6 and L58) overexpressing *ShF5H1* and control plants. (A) acetyl bromide lignin content, (B) lignin monomer composition, (C) S/G ratio, (D) cellulose content. Lowercase letters indicate significant differences (ANOVA followed by Tukey´s test, *p* < .05) among monomers of distinct treatments and capital letters indicate differences in the same treatment (figure B). The vertical bars indicate the standard error of the biological replicates means (*n* = 3 or 4 for T1 lines and control plants, respectively). Each biological replicate was composed of three technical replicates.

Leaves of L4, L6 and L58 transgenic lines were evaluated for lignin content and composition and cellulose content. Lignin content (Figure 2(a)) relative to the cell wall residue (% of cell wall) in *ShF5H1* lines L4, L6 and L58 were 21.4, 21.2 and 20.8%, respectively, and did not differ significantly (*p* = .259) from control plants (24%). Although *ShF5H1* lines showed a tendency of a slight reduction in the lignin content compared to the control (2.2–3.2%), they did not display brownish internodes or midrib leaves as reported for transgenic or mutant plants of other monocot species with reduced lignin content.^31,37,56,77^

In contrast, the lignin composition was clearly altered in leaves under *ShF5H1* overexpression (Figure 2(b)). S monomer increased (*p* ≤ .05) in L4, L6, and L58 lines (55.8%, 93.8%, and 40.3%, respectively) compared to control. G monomer decreased (*p* ≤ .05) in L4, L6, and L58 (42.9%, 45.1%, and 38.6%, respectively) compared to control, which is expected since the carbon flux would be redirected from G to S lignin biosynthesis. S/G ratio (Figure 2(c)) raised significantly (*p* = .00001) in L4, L6, and L58 lines (102%, 161%, and 70%, respectively) compared to control. Unfortunately, we did not measure H unit to check eventual changes in its content, which we believe that did happen. These results demonstrate that *ShF5H1* is crucial for the S-lignin biosynthesis, corroborating investigations using *f5h* mutants, silencing and overexpression of *F5H* homologs in Arabidopsis,^78^ switchgrass,^31^ sorghum,^43^ rice,^42^ barley^44^ and poplar.^30^ Despite the modification of the lignin composition in *ShF5H1* T1 lines, the lignin content did not change significantly, which is similar to the overexpression of *F5H* in sorghum and switchgrass.^31,43^ In contrast, manipulation of lignin metabolism via gene silencing (RNAi) of *F5H*, *COMT*, *4CL*, and *CCoAOMT* and *COMT* talen-mutant resulted in a significant reduction in lignin content^31,35,41,43,56^ (Table S1).

Cellulose content increased significantly only in L6 (*p* ≤ .05), whereas the other lines showed no significant alterations compared to the control (Figure 2(d)).

#### 3.4.2. Phenolic Acids Profile and Total Soluble Phenols

Among the phenolic acids analyzed, the levels of coumaric, quinic acid and chlorogenic acid (CGA) did not show significant changes (*p* > .05). Whereas shikimic acid and total soluble phenols increased in L58, 45% and 31.1%, respectively, compared to the control (data not shown). Further investigations need to be carried out to associate shikimic acid and total soluble phenols increased with the overexpression of *ShF5H1*.

#### 3.4.3. Saccharification Efficiency

**Figure 3.**
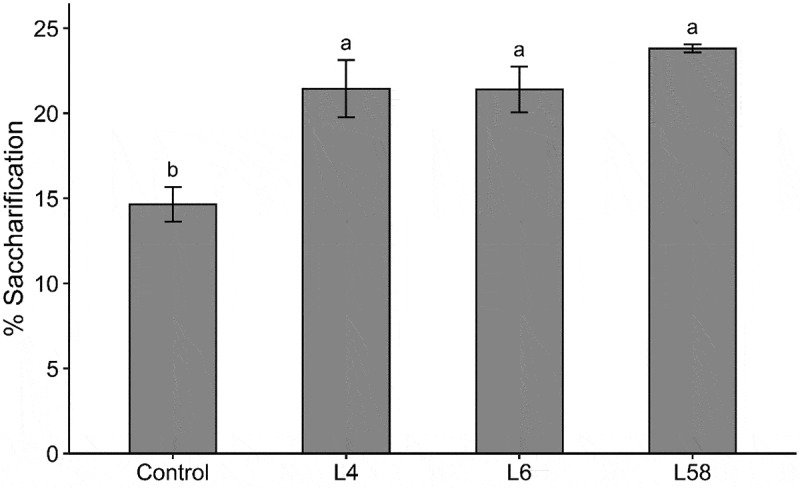
Saccharification efficiency analysis of leaves from T1 transgenic lines (L4, L6 and L58) overexpressing *ShF5H1* and control plants. Lowercase letters indicate significant differences (ANOVA followed by Tukey´s test, *p*<.05). The vertical bars indicate the standard error of the means (*n* = 3 or 4 for T1 lines and control plants, respectively). Each biological replicate was composed of three technical replicates.

L4, L6, and L58 showed significantly higher saccharification efficiency compared to the control (p ≤ .05, *i.e*., 21.4%, 21.4%, and 23.8%, respectively) (Figure 3). The efficiency improved 46% (L4 and L6) and 63% (L58) compared to the control. Thus, leaves are a proper target for the overexpression of *ShF5H1*. Our results and others found in the literature on the genetic modification of the lignin metabolism in sugarcane plants and related species are summarized in Table S1.^31,35,37,41,43,44,56,79,80^ The improvement in saccharification efficiency (from 0% to 72%) in sugarcane and related species differ according to the genetic manipulation strategy, the target gene and tissue, digestion time and pre-treatment. The silencing of *4CL* in sugarcane culms submitted to pre-treatment with diluted acid showed the greatest increase in saccharification efficiency (72%).^38^ The only study we found focusing on the genetic modification of grass leaves for 2GE production reports the silencing of the *BAHD* gene,^79^ which showed an increase of up to 24% in sugarcane saccharification efficiency (Table S1). Rather than targeting lignin metabolism, *BAHD* gene silencing affects the interaction between lignin and hemicelluloses^81^ and.^79^ The consistent improvement in saccharification efficiency of up to 63% (Table S1) in the *ShF5H1* transgenic lines highlights its biotechnological potential to improve the production of 2GE from sugarcane straw.

Based on that, we raised the question: what are the factors contributing to improving saccharification efficiency in *ShF5H1* transgenic lines leaves. The increase in the S/G ratio may be a relevant factor, as this was the convergent and consistent aspect of all transgenic lines with more efficient saccharification we found here. Although lignin content plays a key role in saccharification recalcitrance in grass biomass,^82^ the slight reduction of lignin in *ShF5H1* lines seems less relevant than S-rich lignin. An in-depth examination of all previous reporters cited in Table S1 shows that the saccharification efficiency improvement was associated with lignin content reduction (above 3.9%). Indeed, the *ShF5H1* lines showed only 2.3–3.3% of lignin reduction and greatly impacted saccharification improvement (46–63%). Other investigated parameters, such as the contents of cellulose, hemicellulose, and phenolic compounds showed an unclear relation to the improved saccharification.

Saccharification in sugarcane leaves is more efficient than in culm or bagasse.^26,83,84^ This would partially explain the better efficiency in cellulose digestibility when lignin content is lower, as observed in leaves (13%) when compared with culms.^26^ However, in other reports, the lignin content was similar between the distinct raw materials, straw and bagasse.^83,84^ It should also be considered that S-rich lignin is favorable not only for the deconstruction of lignin but also for the cohesive maintenance of the interaction between the lignin and xylan (hemicellulose) matrix.^26^ Such a dichotomy may partially explain, for example, that the silencing of *COMT* and *F5H* favors saccharification in sugarcane due to the decrease in lignin and also the reduction in the S/G ratio.^35,41,56^ It is known that the degree of cellulose crystallinity,^85^ binding of cell wall components,^79,86^ plant tissue specificity, and physiological conditions^87^ impact cell wall recalcitrance.

### 3.5. ShF5h1, ShCOMT *and* ShCcoaomt1 *Expression Analysis of Leaves*

**Figure 4.**
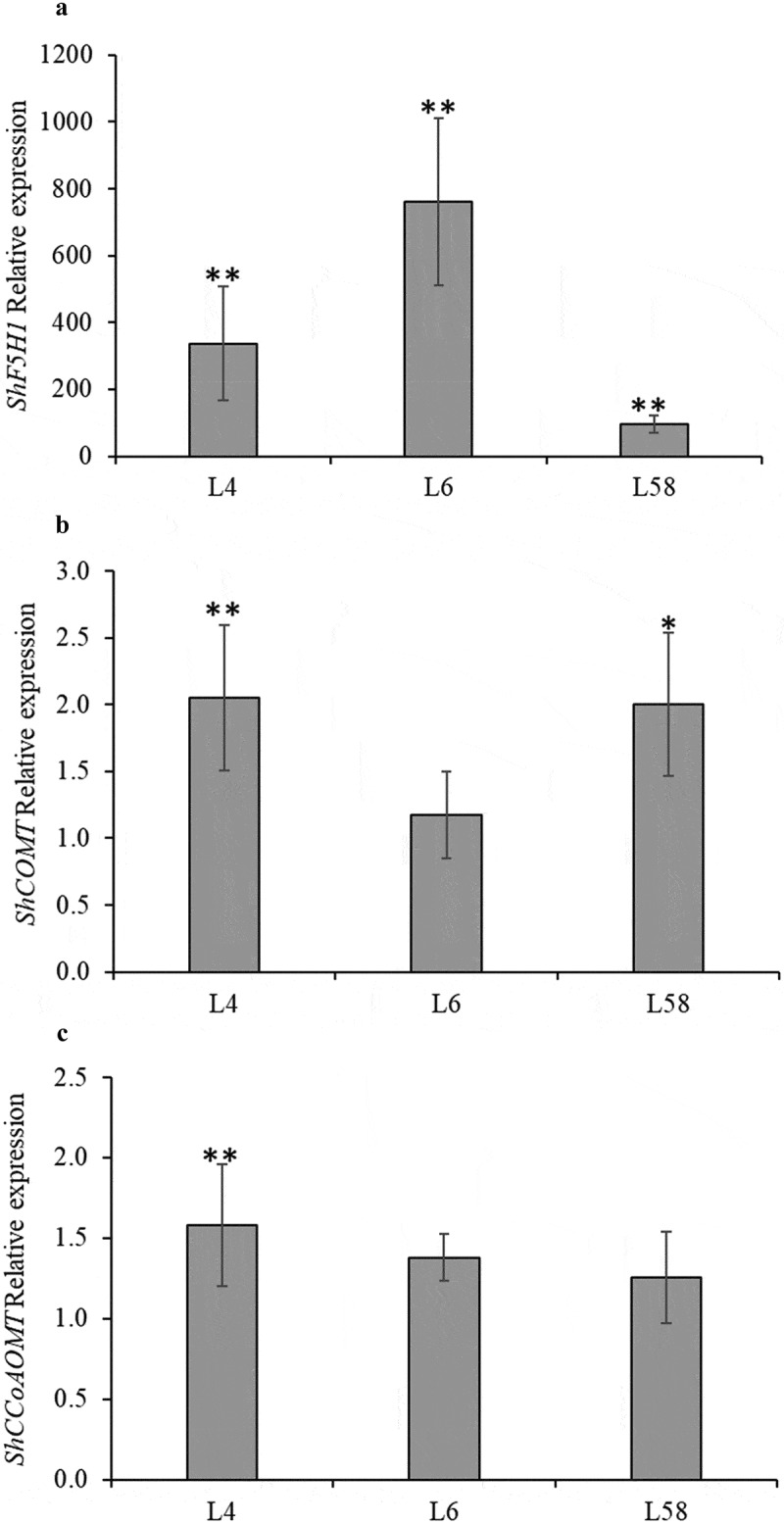
Relative expression of (A) *ShF5H1*, (B) *ShCOMT*, and (C) *ShCcoAOMT* in leaves from T1 transgenic lines (L4, L6, and L58) overexpressing *ShF5H1* compared to the control plants (calibrator). The vertical bars indicate the standard error of the biological replicates means (*n* = 3). Each biological replicate was composed of three technical replicates. One and two asterisks on the top of the bars indicate significant differential expression, *p*≤.005 and *p*≤.001, respectively (REST © software).

The relative expression of *ShF5H1*, *ShCOMT*, and *ShCCoAOMT1* were assessed to better understand S and G lignin monomer biosynthesis in the leaves of T1 lines (Figure 4). *ShF5H1* transcript levels in L4, L6, and L58 showed 337-, 761-, and 95-fold up-regulation (p = .000), respectively, compared to the control (Figure 4(a)). The expression levels of *ShCOMT* were up-regulated in L4 (p = .001) and L58 (p = .005) (2.1 and 2.0-fold higher than the control, respectively) (Figure 4(b)). *ShCCoAOMT1*, which leads to the G monomer biosynthesis branch, displayed a significant and slightly differential expression in L4 (p = .001) (1.6-fold higher than the control) (Figure 4(c)). Interestingly, *ShCOMT* and *ShCCoAOMT1* showed no significant differential expression between L6 (the highest *ShF5H1* transcript level) and the control. However, there is a noticeable trend toward increased expression of *ShCOMT* in transgenic lines. These results suggest that *ShF5H1* overexpression affects differently the expression of *ShCOMT* and *ShCCoAOMT1* genes in sugarcane. Accordingly, *F5H* overexpression in sorghum did not modify the expression profile of lignin pathway enzymes, such as *SbC3H, SbCCoAOMT1, SbCCR*, and *SbCOMT*. Only *Sb4CL* was more expressed in *F5H* transgenic plants than in the control.^43^

### 3.6. ShF5h1 *Overexpression Had a Lower Impact on the 8^th^ Internode Compared to Leaves*

**Figure 5.**
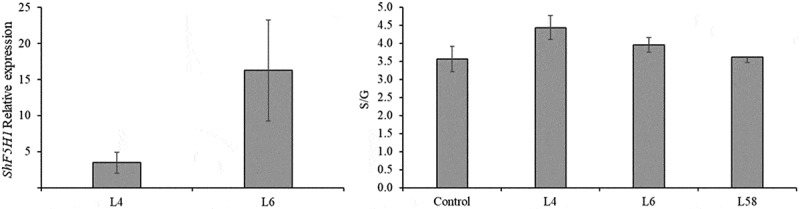
Characterization of the 8^th^ culm internode from T1 transgenic lines (L4, L6 and L58) overexpressing *ShF5H1* and control plants. (A) Relative expression of *ShF5H1*. The vertical bars indicate the standard error of the technical replicates means (*n* = 3). (B) S/G ratio. The vertical bars indicate the standard error of the biological replicates means (*n* = 3 or 4 for T1 lines and control plants, respectively). Each biological replicate was composed of three technical replicates. No statistically significant difference was found between the control and transgenic lines (ANOVA followed by Tukey´s test).

Although *ShF5H1* was up-regulated in the 8^th^ internode of L4 and L6 compared to control (3.5 and 16.2-fold change, respectively) (Figure 5(a)), this was not followed by a real increase in the S/G ratio in culms (Figure 5(b)). In addition, no evident differences in the accumulation patterns of S-lignin were shown in L6 grown in the field compared to the control by anatomical analyses using the Mäule tests, which is a histochemical detection that distinguishes S and G lignins (Figure S6). This can be explained by the fact that the level of the desired end-product can be limited by more than one enzymatic activity in the metabolic pathway. For example, in transgenic Arabidopsis overexpressing *F5H*, COMT appears to be the limiting factor for S biosynthesis.

COMT cannot convert 5-hydroxyconiferyl aldehyde to synapyl-aldehyde at the same rate that F5H overexpressing plants produce 5-hydroxyconiferyl aldehyde.^88^ In contrast, rice mutants generated through CRISPR/Cas9 technology, named as OsCAld5H1-KO, lack the ability to produce OsF5H1.^20^ These mutants even showed the ability to synthesize S-unit monomers from both culm and leaf sheath cell walls. This observation strongly indicates the presence of an alternative, F5H-independent pathway. It is worth noting that the activity of this putative pathway may potentially attenuate the impact of F5H overexpression in sugarcane culm lignin composition. Or yet, differential *ShF5H1* expression between transgenic lines and control did not reach the biological threshold to impact significantly S synthesis, as supported by the S/G and anatomical analyses. The use of a stronger and culm-specific promoter to control *F5H* expression could be an interesting alternative to increase the expression level of this gene and consequently increase the S/G ratio in this tissue. It remains to be elucidated whether an increased S/G ratio in culm would positively affect the efficiency of saccharification, as many known and unknown factors can affect this process.^26,87^

Our results showed that changes in the lignin composition were more pronounced in the leaves than in the culms of transgenic lines, which is expected since the control plants exhibited a higher S/G ratio (Figures 2(c) and 5(b)) and *ShF5H* expression (Figure S7) in the culm (8^th^ internode) compared to the leaf (7.4-fold and 15.6-fold, respectively). The S/G ratios found in control SP80–3280 plants are consistent with other sugarcane varieties showing values ranging from 0.7 to 2.2 in bagasse and culm and from 0.3 to 1.1 in leaves and straw.^26,87,89^

## 4. Conclusions

The overexpression of *F5H* in sugarcane promoted a marked increase in the S/G ratio and saccharification efficiency in the leaves. Few studies so far have explored *F5H* overexpression as a strategy to improve saccharification efficiency. Here, we demonstrated the potential of this strategy in sugarcane for the first time, revealing a strong candidate to become an elite commercial variety. L6 transgenic line showed the best performance, considering the higher saccharification efficiency without alterations in growth and sucrose production under greenhouse conditions. Because productivity has low heritability with high environmental and year effects, the transgenic lines L6, L4, and L58 were taken to field experiments to evaluate technological and agronomic traits for at least three consecutive harvests.

In summary, we demonstrated that *F5H* overexpression significantly improved cellulose conversion to glucose in leaves, opening a new perspective for biomass modification in sugarcane and other bioenergetic crops. The results suggest that fine-tuning the *F5H* expression in specific tissues, like in leaves, may promote better digestibility of residual field biomass without causing penalty in the production of sugar and biofuel.

## Supplementary Material

Supplementary materiaL_GMCF.docx
