# Supplementary material for: ShF5H1 overexpression increases syringyl lignin and improves saccharification in sugarcane leaves
Source: GM Crops Food. 2024 Mar 20;15(1):67–84. doi: 10.1080/21645698.2024.2325181 (PMC10956634; doi:10.1080/21645698.2024.2325181)
Supplement: Supplementary materiaL_GMCF.docx [file KGMC_A_2325181_SM7891.docx]

***ShF5H1* overexpression increases syringyl lignin and improves saccharification in sugarcane leaves**

**Juan Pablo Portilla Llerena^1,2^, Eduardo Kiyota^1^, Fernanda Raquel Camilo dos Santos^3^, Julio C. Garcia^3^, Rodrigo Faleiro de Lima^1^, Juliana Lischka Sampaio Mayer^1^, Michael dos Santos Brito^3,4^, Paulo Mazzafera^1,5^, Silvana Creste^3,6^, Paula Macedo Nobile^3^***

**^1^**Department of Plant Biology, Institute of Biology, University of Campinas, Campinas-SP, Brazil

**^2^**Academic Department of Biology, Professional and Academic School of Biology, Universidad Nacional de San Agustín de Arequipa, Arequipa, Perú.

**^3^**Centro de Cana, Instituto Agronômico (IAC), Ribeirão Preto, SP, Brazil

**^4^**Institute of Science and Technology, Federal University of São Paulo, São José dos Campos, Brazil

**^5^**Department of Crop Science, College of Agriculture “Luiz de Queiroz” – ESALQ, University of São Paulo - USP, Piracicaba-SP, Brazil

**^6^**Departamento de Genética, Faculdade de Medicina de Ribeirão Preto, Universidade de São Paulo, Ribeirão Preto, SP, Brazil

*****Corresponding author:

Paula Macedo Nobile

paulamanobile@gmail.com

**Supplementary material for:** Llerena et al. *ShF5H1* overexpression increases syringyl lignin and improves saccharification in sugarcane leaves.

# Section 1. Tables

##

## **Table S1.** Literature data on transgenic sugarcane plants and related species genetically modified for lignin. The table indicates plant species, plant tissue (feedstock), genetic manipulation strategy (overexpression, suppression, mutation), target gene, lignin content, S/G ratio, and saccharification efficiency concerning non-transgenic or mutant control plants (wild type and/or tissue culture control plants).

| **Plant** | **Feedstock** | **Genetic Manipulation** | **Target** | **Pretreatment** | **Digestion Time** | **Lignin content** | **S/G** | **Sac %**** | **Reference** |
| --- | --- | --- | --- | --- | --- | --- | --- | --- | --- |
| Sugarcane | Leaves | overexpression | *F5H1* | NA | 120 h | Unchanged | Increase | 63% | Present work |
| Sugarcane | Bagasse | suppression | *F5H1* | mild* | 72 h | Unchanged | Reduction | 31% | Bewg et al. 2016 |
| Sugarcane | Bagasse | suppression | *COMT* | mild* | 72 h | Reduction | Reduction | 51% | Bewg et al. 2016 |
| Sugarcane | Bagasse | suppression | *CCoAOMT* | mild* | 72 h | Reduction | Unchanged | 28% | Bewg et al. 2016 |
| Sugarcane | Culm | suppression | *COMT* | NA | 120 h | Reduction | Reduction | 29% | Jung et al 2012 |
| Sugarcane | Culm | suppression | *COMT* | diluted-acid | 120 h | Reduction | Reduction | 34% | Jung et al 2012 |
| Sugarcane | Culm | suppression | *COMT* | diluted-acid | 72 h | Reduction | Reduction | 23% | Jung et al 2013 |
| Sugarcane | Culm | suppression | *COMT* | diluted-acid | 72 h | Reduction | Reduction | 32% | Jung et al 2013 |
| Sugarcane | Bagasse | Talen- mutant | *COMT* | diluted-acid | 72 h | Reduction | Reduction | 44% | Kannan et al 2018/ Jung and Altpeter 2016 |
| Sugarcane | Stalks | suppression | *4CL* | diluted-acid | 120 h | Reduction | Increase | 72% | Jung et al 2016 |
| Sugarcane | Straw | suppression | *BAHD* | organosolv | 48h | Unchanged | Unchanged | 24% | Souza et al 2019 |
| Barley | Straw | suppression | *F5H1* | hot water | 96 h | Unchanged | Reduction | Unchange | Shafiei et al 2023 |
| Sorghum | Mature stover | overexpression | *F5H1* | NA | NA | Unchanged | Increase | NA | Tetreault et al 2020 |
| Sorghum | Mature stover | overexpression/mutant | *F5H/COMT* | NA | NA | Reduction | Increase | NA | Tetreault et al 2020 |
| Switchgrass | Young culm | suppression | *COMT* | NA | NI | Reduction | Reduction | 26% | Wu et al 2019 |
| Switchgrass | Young culm | suppression | *F5H1* | NA | NI | Unchanged | Reduction | 4% | Wu et al 2019 |
| Switchgrass | Young culm | overexpression | *F5H1* | NA | NI | Unchanged | Increase | 1% | Wu et al 2019 |
| Switchgrass | Young culm | suppression/suppression | *F5H1/COMT* | NA | NI | Reduction | Reduction | 59% | Wu et al 2019 |
| Switchgrass | Young culm | overexpression/suppression | *F5H1/COMT* | NA | NI | Reduction | Increase | 41% | Wu et al 2019 |

*mild pretreatment of 1% (w/w) sulfuric acid, ** Saccharification improvement percentage in relation to control (counterpart non-genetically modified), NA – not analyzed, NI – not informed

## **Table S2.** Primer pairs used for *ShF5H1* cloning, gene expression analysis and confirmation of transgene integration into genomic DNA.

| **Gene** | **Forward 5´-->3´** | **Reverse 5´-->3´** | **Amplicon (bp)** |
| --- | --- | --- | --- |
| **Cloning** | | | |
| *ShF5H1*_CDS | ATGGCGGCCGTGGCCAAGAT | TCAGTACAAGGGGCAGTTGA | 1584 |
| *ShF5H1*_GTW | AGCAGGCTTCACCATGGCGGCCGTGGCCAAGAT | GAAAGCTGGGTCTCAGTACAAGGGGCAGTTGA | 1609 |
| attB1-2 | GGGGACAAGTTTGTACAAAAAAGCAGGCT | GGGGACCACTTTGTACAAGAAAGCTGGG | 1641 |
| pC4H2070 | TGACTGGTTGGAGATAGAGA | TGCCGCCGCCGCTGCTCTGCT | 2070 |
| pC4H2070_L4R1 | caactttgtatagaaaagTTGTGACTGGTTGGAGATAGAGAGAGAT | aacttttttgtacaaacTTGTGCCGCCGCCGCTGCTCT | 2015 |
| **Expression analysis** | | | |
| *ShCOMT* (RT-qPCR) | GAGGACAAGGACGGCAAGTA | ACCGCGTCCTTGAGGTAGTA | 154 |
| *ShF5H1* (RT-qPCR) | CTTCCTCAAGTGCGTCATCA | GCAGTCGTCGGCAGTCTC | 82 |
| *ShCCoAOMT1* (RT-qPCR) | ACGCCGACAAGGACAACTAC | GCGGTAGAAGCGGATGTACT | 151 |
| *ShGAPDH* (RT-qPCR) | TTGGTTTCCACTGACTTCGTT- | CTGTAGCCCCACTCGTTGT | 122 |
| **Confirmation of transgene integration into genomic DNA** | | | |
| *ShF5H1­_T35S* | GCAGGTCACTGGATTTTGGT | GATGGCCCACACGTTGAT | 758 |

## **Table S3.** Composition of Murashige and Skoog (MS) Basal Medium per litre (L^-1^) used for: 1. *calli* induction (SCIM_3_), 2. cell multiplication and selection, 3. shoot regeneration and 4. elongation and rooting.

| **Components** | **1** | **2** | **3** | **4** |
| --- | --- | --- | --- | --- |
| MS basal salts powder mixture | 4,33 g | 4,33 g | 4,33 g | 4,33 g |
| Vitamins MS 1000x | 1 mL | 1 mL | 1 mL | 1 mL |
| Sucrose | 20 g | 20 g | 20 g | 20 g |
| L-cysteine | 100 mg | 100 mg |  |  |
| Citric acid | 150 mg | 150 mg |  |  |
| Myo-inositol | 100 mg | 100 mg |  |  |
| 2,4-D^a^ | 3 mg | 3 mg |  |  |
| BAP^b^ |  |  | 1 mg |  |
| Phytagel | 3 g |  |  |  |
| Agarose |  | 6 g |  |  |
| Agar |  |  | 7 g | 7 g |
| Meropenem |  | 20 mg | 20 mg | 20 mg |
| G418 geneticin |  | 50 mg | 40 mg | 30 mg |

## ^a^2,4-dichlorophenoxyacetic acid

## ^b^benzylaminopurine

## **Table S4.** Relative transgene copy number. The CT data were extracted from Applied Biosystems StepOnePlus System. The relative transgene copy number (RTCN) was estimate for each event (L1 to L60) compared to the transgenic line (L31) with higher CT value (CT =26.2), calculated according to the formula RTCN=2^CTLn-CTL31^. The classification of relative copy number was high (RTCN >24); intermediary (23> RTCN >12) and low (RTCN<12).

| Trangenic events C4H:F5H | | Cт | Δ_CT_  (CT_Ln_-CT_L31_) | Relative transgene copy number  (2^CTLn-CTL31^) | |
| --- | --- | --- | --- | --- | --- |
| L | 11 | 19.0 | 7.2 | 145 | high |
| L | 49 | 19.8 | 6.4 | 84 | high |
| L | 16 | 19.9 | 6.3 | 76 | high |
| L | 42 | 20.3 | 5.9 | 61 | high |
| L | 30 | 20.3 | 5.9 | 60 | high |
| L | 17 | 20.9 | 5.2 | 38 | high |
| L | 6 | 21.0 | 5.2 | 37 | high |
| L | 32 | 21.0 | 5.1 | 35 | high |
| L | 4 | 21.1 | 5.1 | 34 | high |
| L | 10 | 21.1 | 5.1 | 33 | high |
| L | 19 | 21.6 | 4.6 | 25 | high |
| L | 28 | 21.6 | 4.6 | 24 | high |
| L | 47 | 21.7 | 4.5 | 22 | intermediary |
| L | 18 | 21.7 | 4.4 | 22 | intermediary |
| L | 57 | 22.0 | 4.1 | 17 | intermediary |
| L | 58 | 22.1 | 4.1 | 17 | intermediary |
| L | 13 | 22.1 | 4.1 | 17 | intermediary |
| L | 51 | 22.1 | 4.1 | 17 | intermediary |
| L | 56 | 22.3 | 3.9 | 14 | intermediary |
| L | 2 | 22.4 | 3.8 | 14 | intermediary |
| L | 1 | 22.4 | 3.7 | 13 | intermediary |
| L | 22 | 22.4 | 3.7 | 13 | intermediary |
| L | 25 | 22.5 | 3.7 | 13 | intermediary |
| L | 20 | 22.5 | 3.7 | 13 | intermediary |
| L | 36 | 22.6 | 3.6 | 12 | low |
| L | 55 | 22.7 | 3.5 | 11 | low |
| L | 53 | 22.8 | 3.4 | 11 | low |
| L | 27 | 22.8 | 3.4 | 11 | low |
| L | 26 | 22.9 | 3.3 | 10 | low |
| L | 45 | 22.9 | 3.3 | 10 | low |
| L | 7 | 22.9 | 3.3 | 10 | low |
| L | 46 | 22.9 | 3.2 | 9 | low |
| L | 52 | 22.9 | 3.2 | 9 | low |
| L | 21 | 23.0 | 3.2 | 9 | low |
| L | 29 | 23.3 | 2.9 | 7 | low |
| L | 35 | 23.3 | 2.9 | 7 | low |
| L | 38 | 23.3 | 2.9 | 7 | low |
| L | 5 | 23.3 | 2.8 | 7 | low |
| L | 60 | 23.6 | 2.6 | 6 | low |
| L | 24 | 23.7 | 2.5 | 6 | low |
| L | 23 | 23.8 | 2.4 | 5 | low |
| L | 43 | 23.9 | 2.3 | 5 | low |
| L | 8 | 24.0 | 2.2 | 5 | low |
| L | 59 | 24.1 | 2.0 | 4 | low |
| L | 9 | 24.1 | 2.0 | 4 | low |
| L | 40 | 24.2 | 2.0 | 4 | low |
| L | 54 | 24.3 | 1.8 | 4 | low |
| L | 34 | 24.4 | 1.8 | 4 | low |
| L | 33 | 24.5 | 1.7 | 3 | low |
| L | 3 | 24.6 | 1.6 | 3 | low |
| L | 14 | 24.6 | 1.6 | 3 | low |
| L | 37 | 24.8 | 1.4 | 3 | low |
| L | 39 | 24.9 | 1.3 | 2 | low |
| L | 12 | 25.4 | 0.8 | 2 | low |
| L | 15 | 25.7 | 0.5 | 1 | low |
| L | 48 | 25.8 | 0.4 | 1 | low |
| L | 44 | 25.8 | 0.4 | 1 | low |
| L | 50 | 25.9 | 0.3 | 1 | low |
| L | 31 | 26.2 | 0.0 | 1 | low |

# Section 2. Figures

## **Figure S1**. Rice C4H promoter alignment**.** Genomic sequences from GenBank (LOC_Os05g25640, Phytozome (Chr5), and DNA cloning from *Oryza sativa* var. Nipporbare were used for pC4H-F5H construction.

## **Figure S2.** Schematic representation of the T-DNA expression cassette from the binary vector pJFNPT_pOsC4H:ShF5H1. pOsC4H, rice C4H promoter; ShF5H1, sugarcane codon DNA sequence of the *F5H1* gene, T35S, CaMV 35S terminator.


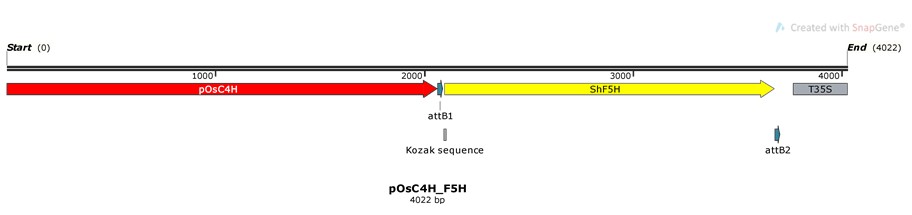


## **Figure S3.** Phylogenetic tree of the F5H protein family and related sequences. Phylogenetic analyses of the ShF5H1 and F5H protein sequences from sorghum (Sb), rice (Os), maize (Zm), *Arabidopsis thaliana* (At), and *Panicum virgatum* (Pavir), were generated using the Maximum Likelihood method (ML) in MEGA version 10.2.4. Bootstrap values (100 replicates) are shown for nodes in the tree. Asterisk symbols indicate the functionally characterized proteins through overexpression and/or silencing.


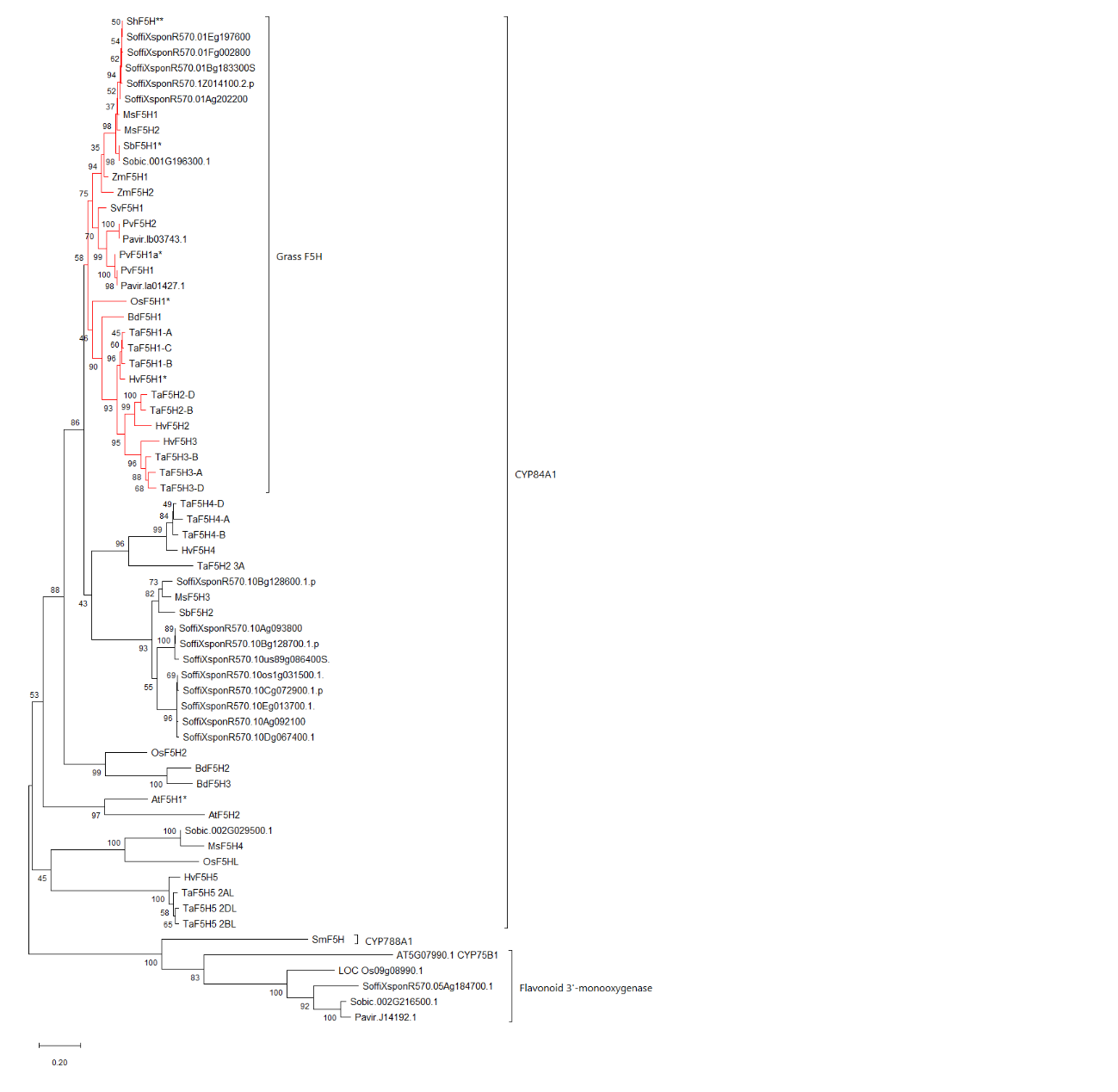


## **Figure S4.** Transgenic events (T0) of the sugarcane hybrid SP80-3280 harboring *ShF5H1* overexpression and their respective controls. (A) 1-3 - regenerated shoots in MS selective medium containing the antibiotic geneticin (G418), 4 - control regenerated shoots in MS medium without antibiotic, 5 - control negative in MS selective medium with G418. (B) - Detail of a regenerated shoot (arrow). (C) - Control (no selective medium) and transgenic events (selective medium) seedlings.


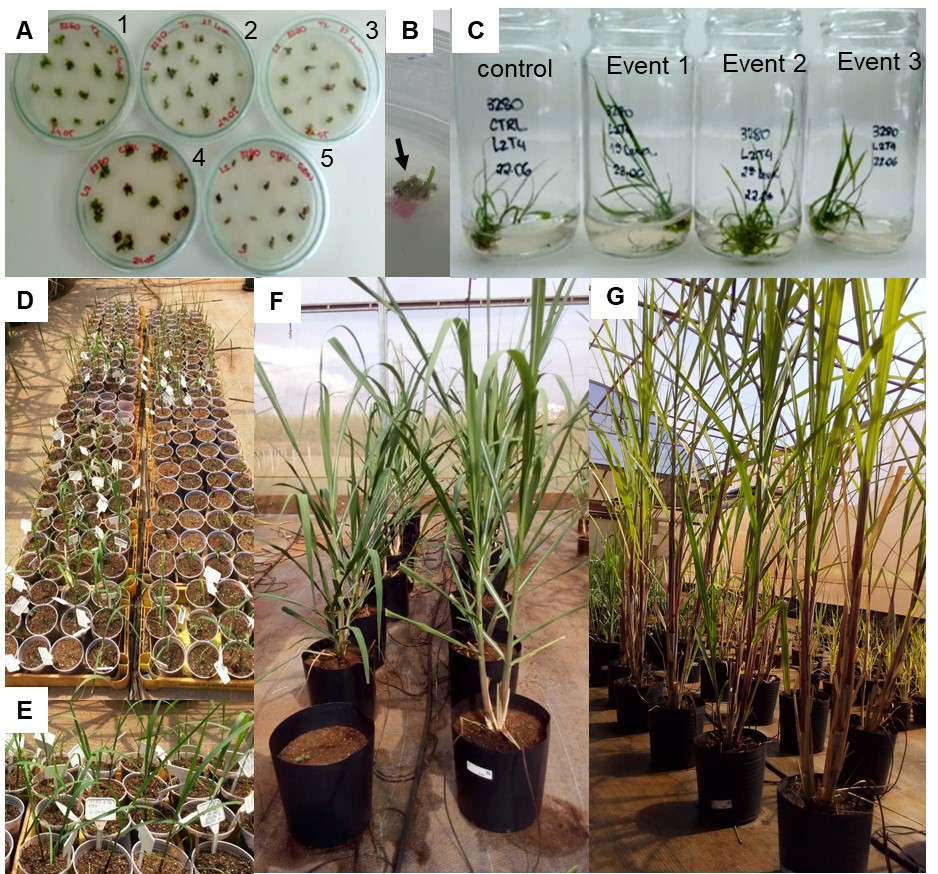


## **Figure S5**. Screening of the *ShF5H*1 transgenic events (T0). (A) - Relative expression of *ShF5H1* transcripts in leaves of 31 events compared to three control plants. (B) - Lignin content analysis of syringil /guaiacyl (S/G) ratio in leaves of nine events selected from the transcriptional analysis and three controls.


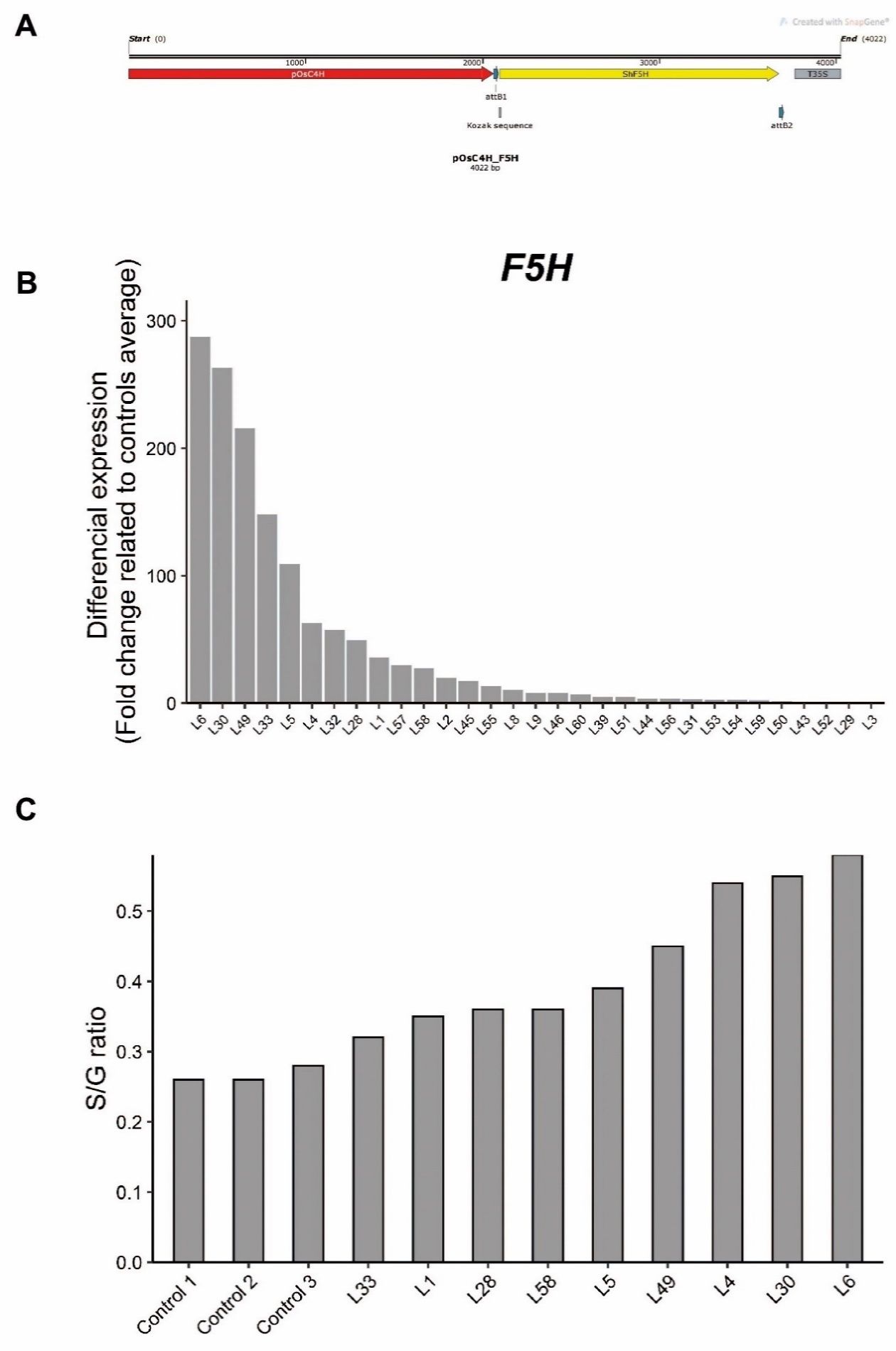


B

A

**Figure S6.** Cross-sectional views of the culm (A – P) of SP80-3280 variety (*Saccharum spp*). Control (wild-type) and *ShF5H* transgenic line L6. Culm sections in the control (A) and transgenic (C) samples of sugarcane demonstrate the presence of vascular bundles interspersed with filling parenchyma, with emphasis in B and D on a single vascular bundle, highlighting the width of the fibrous vascular sheath. Culm sections of sugarcane peripherical region post-reaction with the Mäule reagent in control (E, F) and transgenic samples (G, H), respectively. Note the difference in the presence, primarily, of G lignin, indicated by the yellow arrows in F and H. In G, the presence of lignified parenchyma in the peripheral region of transgenic samples is observed. Stem sections of sugarcane pith region post-reaction with the Mäule reagent in control (I, J) and transgenic (K, L) samples. Note the presence of S lignin on the fiber of vascular bundle (J, L) indicated by the yellow arrows. (M, N; O, P). Culm sections of sugarcane post-reaction with phloroglucinol highlight the presence of lignin mainly in the vascular bundles and filling parenchyma. Note the difference in the lignification process of the fibers that compose the vascular sheath on transgenic samples in N and P (P). Fp = filling parenchyma; Fi = fiber; Mx = vessel from metaxylem; Ph = floem; Px = vessel from protoxylem; Vbs = vascular bundle sheath; Vb = vascular bundle. Bars: 500µm = (A, C, I, K, M, O); 200µm = (E, F, G, H,); 100µm = (B, D, J, L); 50µm = (N, P).


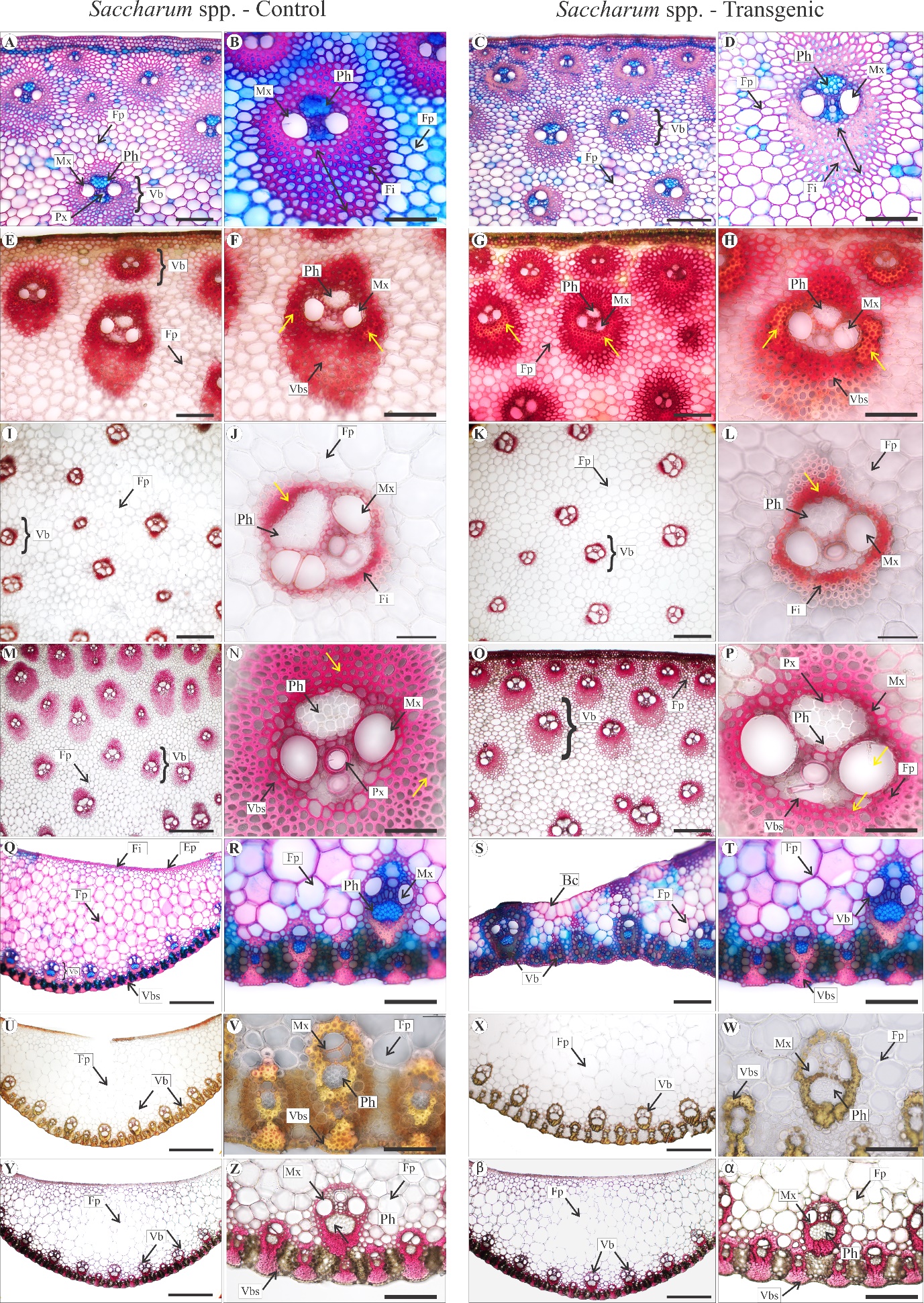


In both analyzed materials, the control (wild type) and transgenic L6, the culm is formed by a lignified unstratified epidermis, followed by one to two layers of parenchymal cells. The peripheral region of the culm in both samples consists of larger diameter vascular bundles, composed of primary xylem and phloem surrounded by a fibrous vascular sheath (Figures S6A and S6C), which, in the control (Figure S6B), appeared larger compared to the transgenic ones (Figure S6D). The Mäule tests revealed the presence of S lignin in the layers beneath the epidermis, in the cell wall of the cells of the vascular bundles, and in the cell wall of the fundamental parenchyma of the peripheral region (Figures S6E, S6F, S6G and S6H). Meanwhile, lignin G was only visualized in the fibers of the vascular sheath (Figures S6F and S6H, yellow arrows). In control sample, S lignin is predominantly present in the walls of vessel elements and in the fibers that make up the vascular sheath (Figure S6E). On the other hand, G lignin was observed in some fibrous cells near the vessel elements, mainly (Figure S6F). In the L6 transgenic sample, G lignin was detected in various cells of the vascular sheath, while S was present in the walls of vessel elements, in the outermost fibers of the sheath, in the layers beneath the epidermis, and in the fundamental parenchyma (Figures S6G and S6H). In the medullary region, the vascular bundles were smaller, with little vascular sheath, immersed in the parenchymal filling tissue (Figures S6I and S6K). The Mäule test revealed the presence of only the S lignin on the fibers near the vessel elements in control (Figure S6J, yellow arrows) and transgenic (Figure S6L, yellow arrows) samples. The phloroglucinol tests revealed the presence of lignin in the same cells indicated in the Mäule test (Figures S6M, S6N, S6O and S6P). However, differences in the degree of lignification of the fibers composing the vascular sheath were observed (Figures S6N and S6P). The fibers more deeply stained in red were already lignified, usually close to the vessel elements, while the outermost fibers showed a pink-purple coloration, indicating incomplete lignification (Figures S6N and S6P, yellow arrows). For both control and L6 samples, no significant difference was observed, only a higher degree of lignification in the cells of the fundamental parenchyma of the peripheral region of the culm in transgenic sections (Figure S6O), probably due to developmental differences in samples that were collected in an uncontrolled field condition.

**Figure S7**. Comparison of *ShF5H1* expression between the culm (8^th^ internode) and leaf. Culms and leaves were sampled from two non-transformed plants (Control) that were 9-months-old (Material and Methods, topic 2.2). The relative quantification method (Pfaffl, 2001) was calculated using *ShGAPDH* as reference gene. The *ShF5H* relative expression was in average 15.6-fold higher in the 8^th^ internode compared to leaves. The vertical bars indicate the standard error of two biological replicates.

Reference

PFAFFL, Michael W. A new mathematical model for relative quantification in real-time RT–PCR. Nucleic acids research, v. 29, n. 9, p. e45-e45, 2001.
